# Influence of the “five‑in‑one” mode on complications and compliance behaviors of patients with ureteral calculi after minimally invasive surgery 

**DOI:** 10.20452/wiitm.2024.17893

**Published:** 2024-07-31

**Authors:** Xixian Ou, Xuejing Wang

**Affiliations:** The First Affiliated Hospital of Nanjing Medical University, Nanjing, Jiangsu Province, China

**Keywords:** compliance, complication, minimally invasive surgery, mode, ureteral calculi

## Abstract

**INTRODUCTION:**

As a clinically common urinary system disease, ureteral calculus mainly manifests as hematuria and colicky pain.

**AIM:**

We aimed to explore the influence of the “five‑in‑one” mode on complications and compliance behaviors of patients with ureteral calculi after minimally invasive surgery.

**MATERIALS AND METHODS:**

A total of 92 participants were enrolled from among the patients hospitalized for ureteral calculi and treated with minimally invasive surgery between April 2022 and April 2023. The patients were randomized into a control group (n = 46) and an experimental group (n = 46). The control group received rehabilitation guidance under routine nursing mode, while the experimental group was provided with the “five‑in‑one” nursing mode to strengthen communication in all aspects, in addition to synergistic therapy.

**RESULTS:**

The self‑rating depression scale scores declined in both groups after treatment in comparison with those before treatment (*P* <0.05), and the postoperative score was significantly lower in the experimental group (*P* <0.05). The experimental group had also decreased Visual Analog Scale scores in all time periods (1, 3, and 7 d) after treatment (*P* <0.05), and significantly elevated total nursing satisfaction rate, as compared with that of the control group (97.82% vs 82.6%; *P* <0.05).

**CONCLUSIONS:**

The “five‑in‑one” mode can enhance patient confidence in rehabilitation and improve their quality of life and satisfaction with nursing.

## INTRODUCTION

As a clinically common urinary system disease, ureteral calculus mainly manifests with hematuria and colicky pain, often accompanied by urinary tract infection, urinary tract obstruction, and other complications, which may even impair the renal function and seriously jeopardize physical and mental safety of the patients, which is why timely calculus removal is necessary.^1^ Surgery is the major therapeutic method for ureteral calculi removal, and more types of minimally invasive surgeries for ureteral calculi are emerging with the progress and development of medical technology, including flexible ureteroscopy, extracorporeal shock wave lithotripsy, percutaneous nephrolithotomy, and laparoscopy.^2^ Although minimally invasive surgery shortens the hospitalization time, reduces the surgical injury, and alleviates pain, conditions such as noncompliance with treatment, existence of other underlying diseases, and poor recognition of indications may lead to postoperative complications, such as bleeding, ureteral mucosal injury, and infection. Moreover, some patients poorly understand the surgical procedure of calculi removal, and they are extremely prone to develop anxiety, nervousness, and other negative emotions, which are unfavorable to their recovery, prolong the hospitalization time, and increase their distress.^3^ The “five‑in‑one” mode is a novel nursing mode in which doctors, nurses, therapists, patients, and patient family members cooperate with each other. Specifically, the doctors adjust the rehabilitation scheme according to the patient condition, the nurses care for the patients, the therapists instruct the patients to accomplish rehabilitation training, and the patients learn the disease‑related information and comply with the medical orders. Moreover, the patient family members communicate with the patient, observe their recovery, report it to the doctors, accompany the patient, and calm them down if they are too emotional, jointly promoting the recovery.^4^

## AIM

The study intended to investigate the influence of the “five‑in‑one” nursing mode on complications and compliance behaviors in patients with ureteral calculi after minimally invasive surgery, thereby rendering thoughts and references for subsequent nursing approach.

## MATERIALS AND METHODS

General data A total of 92 patients suffering from ureteral calculi, who were hospitalized from April 2022 to April 2023 and underwent minimally invasive surgery, were selected. They were labeled based on the time of admission by a data tagging method, and randomly allocated into the control group (n = 46) and the experimental group (n = 46).

### Inclusion and exclusion criteria

The following inclusion criteria were adopted: 1) no surgery within the past 6 months, 2) no metabolic diseases, 3) no contraindications to minimally invasive surgery, 4) clear consciousness, normal communication ability, no communication disorders, and 5) ability to strictly comply with the treatment protocol provided by the therapists.

The exclusion criteria included: 1) severe organ diseases, 2) mental illnesses, 3) unwillingness to participate in the study on the patient’s or their family’s side, or 4) a small number of residual calculi or incompletely mitigated symptoms.

### Treatment method for the control group

Routine nursing intervention was adopted. Patient information was recorded, medication instructions were formulated for the patients, daily life and dietary patterns of the patients were adjusted, patient mental status was observed, and psychological guidance was offered timely. Meanwhile, the patients were provided with health education, and supervised to comply with medical orders for 7 consecutive days.

### Treatment method for the experimental group

The “five‑in‑one” nursing mode with the steps described below was implemented. The “five‑in‑one” mode team comprised doctors, nurses, professional therapists, patients, and patient family members. All team members were required to convey the patient information in a timely manner, maintain mutual communication, and work together to formulate and promptly adjust the rehabilitation scheme.

First, the doctors were responsible for developing healthy recipes according to the specific conditions of the patients, adjusting their dietary habits, and standardizing their daily life, so as to accelerate recovery. At the same time, the doctors provided the patients and their family members with health education guidance by means of PowerPoint presentations, short videos and so on, and the duration of guidance was flexibly adjusted according to the personal knowledge comprehension ability and concentration of the patients. After that, the doctors verified the efficacy of health education by asking the patients a couple of health–related questions and listening to their answers.

Second, the nurses were required to actively talk with the patients in the process of nursing to alleviate their negative emotions, assist in rehabilitation exercises, supervise the patients to follow the rehabilitation scheme, and advise the family members of poor compliance to supervise and guide such patients.

Third, the therapists developed rehabilitation training methods based on individual recovery speed, and explained the rehabilitation scheme to the patients, so that they could understand the training methods and effects and to improve their compliance. Specifically, the rehabilitation scheme encouraged the patients to drink more water and urinate more to promote metabolism, improved their cognition, so that they could understand the cause of pain and its current status, thus relieving the psychological burden, and inspired the patients to get out of bed to exercise 1–2 times per hour, without clear requirements for the exercise mode. If the patients needed to lie in bed for recovery, their position was adjusted regularly to prevent pressure ulcers and other complications. In the meantime, the therapists instructed the patients to conduct meditation training and relax their body, strengthened the communication with the patients to learn about their actual condition, and discussed and adjusted the rehabilitation scheme with the doctors.

Fourth, the patients were required to strictly follow the rehabilitation scheme and report their pain in a timely manner without concealment.

Fifth, the patient family members should provide encouragement and support for the patients, frequently communicate with them, and alleviate their anxiety. At the same time, they needed to supervise the patients to see if they follow the rehabilitation scheme, actively communicate with the medical staff, and cooperate with each other to speed up the patient recovery. The therapeutic intervention lasted for 7 days after the surgery.

### Evaluation of patient physical status

The physical status of the patients was assessed before the surgery and 7 days after it using the Karnofsky performance status (KPS) score (0–100 points).^5^ A higher KPS score indicated better quality of life (QoL). The KPS scores of both groups were recorded, and the QoL before and after the treatment was compared. In addition, the self‑rating depression scale (SDS) was employed to evaluate mental status of the patients,^6^ with higher SDS score signifying worse mental status.

### Assessment of the pain level

The postoperative pain level was appraised with the Visual Analogue Scale (VAS) at 1, 3, and 7 days after the surgery.^7^ Pain was rated from none to severe on a 0–10 scale. The higher the VAS score, the stronger the pain.

### Evaluation of the complication rate

Complications of minimally invasive surgery for ureteral calculi included infection, bleeding, and ureteral perforation.

### Assessment of patient compliance

A self‑developed compliance scale was utilized to evaluate patient compliance in 5 aspects: medication, rehabilitation exercise, communication, learning, and basic clinical data (including age, calculus diameter and type of surgery), with a full score of 10 points for each aspect and a total score of 0–50 points. Higher score indicated better patient compliance.

### Evaluation of patient satisfaction

The patients were surveyed using a self‑developed questionnaire (0–100 points), mainly involving questions on treatment methods, nursing conditions, personnel attitudes, and time schedules. The score above 90 points was deemed satisfactory, 70–90 points relatively satisfactory, and below 70 points unsatisfactory.

### Statistical analysis 

SPSS 20.0 software (IBM, Armonk, New York, United States) was employed for statistical analysis. The measurement data including KPS, SDS, VAS score, and patient compliance were expressed as mean and SD. The VAS score assessed repeatedly was subjected to repeated‑measures analysis of variance. The count data, such as complication rate and patient satisfaction, were analyzed with the χ^2^ test or the Fisher test, and the *t* test was conducted for comparison between the groups. The *P* value below 0.05 was deemed significant.

### Ethics

This study approved by Jiangsu Provincial People’s Hospital ethics committee (20220645), and written informed consent form was obtained from all patients.

## RESULTS 

### General data

The control group comprised 31 men and 15 women aged 23–59 years, at a mean (SD) age of 45.17 (10.9) years. The calculus diameter ranged from 7.1 mm to 16.7 mm, with a mean (SD) of 11.41 (3.27) mm. As for the type of minimally invasive surgery, 21 patients received extracorporeal shock wave lithotripsy, 13 patients underwent laparoscopy, and 12 patients were treated with flexible ureteroscopy.

The experimental group consisted of 28 men and 18 women aged 21–58 years, at a mean age of 46.26 (11.43) years. The calculus diameter was 6.1 mm to 17.4 mm, with a mean of 12.62 (3.18) mm. Regarding the minimally invasive surgery, extracorporeal shockwave lithotripsy, laparoscopy, and flexible ureteroscopy were performed in 19, 14, and 13 patients, respectively. No significant differences between the groups were observed in the general data covering sex, age, type of minimally invasive surgery, and the size of ureteral calculi.

### Karnofsky performance status and self‑rating de‑ pression scale scores before and after treatment

The scores before treatment showed no differences between the 2 groups (*P* >0.05). The KPS score rose in both groups at 7 days after treatment, as compared with its value before treatment (*P* <0.05), and the increase was significantly greater in the experimental group. The SDS scores declined in both groups at 7 days after treatment vs those before treatment (*P* <0.05), and they were significantly lower in the experimental than those in the control group (TABLE 1).

**TABLE 1:** Karnofsky performance status and self‑rating depression scale scores before and after treatment

Parameter	KPS score		SDS score	
	Before treatment	7 d after treatment	Before treatment	Before 7 d after treatment treatment
Experimental group (n = 46)	62.17 (6.61)	85.23 (8.76)^a^	40.17 (4.12)	24.61 (5.21)^a^
Control group (n = 46)	61.94 (5.97)	74.21 (8.13)^a^	40.26 (4.21)	34.26 (5.17)^a^
t	0.175	6.253	0.103	8.917
p	0.86	<0.001	0.92	<0.001

Data are presented as mean and SD.

a *P* <0.05 vs the same group before treatment

Abbreviations: KPS, Karnofsky performance status; SDS, self‑rating depression scale

Pain level The experimental group exhibited lower VAS scores for pain than the control group for all time periods (1, 3, and 7 d) after treatment (*P* <0.05) (TABLE 2 ).

**TABLE 2:** Visual analogue scale scores of patients after treatment

Parameter	1 d after treatment	3 d after treatment	7 d aftertreatment
Experimental group (n = 46)	3.21 (0.87)	1.49 (0.47)	1.06 (0.32)
Control group (n = 46)	4.03 (1.01)	1.83 (0.61)	1.31 (0.39)
*F*_time_; *P*_time_	*F*_time_ = 4.937; *P*_time_ = 0.007		
*F*_between groups_; *P*_between_	*F*_between groups_ = 350.473; *P*_between groups _<0.001		
*F*_interaction_; *P*_interaction_	*F*_interaction_ = 34.856; *P*_interaction _<0.001		

Data are presented as mean and SD.

### Complication rates

A lower postoperative complication rate was obtained in the experimental group than in the control group (4.34% vs 17.39%; *P* <0.05) (TABLE 3).

**TABLE 3:** Postoperative complication rate

Parameter	Infection	Bleeding	Ureteral perforation	Total complication rate
Experimental group (n = 46)	0	1 (2.17)	1 (2.17)	2 (4.34)
Control group (n = 46)	3 (6.52)	4 (8.69)	1 (2.17)	8 (17.39)
χ2	–	–	–	4.039
P	–	–	–	0.044

Data are presented as number (percentage) of patients.

### Patient compliance

After intervention, the experimental group displayed significantly improved compliance with all aspects of the rehabilitation treatment, as compared with the control group (TABLE 4 ).

**TABLE 4:** Patient compliance score

Parameter	Medication	Rehabilitation exercise	Communication	Learning	Basic tests
Experimental group (n = 46)	7.26 (1.08)	6.94 (1.28)	8.71 (0.84)	5.51 (0.89)	8.62 (0.71)
Control group (n = 46)	6.73 (1.24)	5.74 (0.54)	6.87 (0.97)	4.01 (0.53)	7.14 (0.83)
t	2.186	5.858	9.725	9.821	9.19
P	0.03	<0.001	<0.001	<0.001	<0.001

Data are presented as mean and SD.

### Patient satisfaction with nursing

Total nursing satisfaction rate was markedly higher in the experimental than in the control group (97.82% vs 82.60%; *P* <0.05) (TABLE 5).

**TABLE 5:** Patient satisfaction with nursing

Parameter	Satisfied	Relatively satisfied	Dissatisfied	Total satisfaction rate
Experimental group (n = 46)	33 (71.73)	12 (26.08)	1 (2.17)	45 (97.82)
Control group (n = 46)	24 (52.17)	14 (30.43)	8 (17.39)	38 (82.6)
P	–	–	–	0.03

Data are presented as number (percentage) of patients.

## DISCUSSION

The leading predisposing factors for ureteral calculi include improper diet, metabolic disorders caused by chaotic work and rest schedules, and drinking too little water.^8^^;^^9^ Clinical symptoms of ureteral calculi include hematuria, vomiting, pain, etc. With inappropriate treatment methods or delayed treatment the disease may extremely easily progress into sepsis, acute pyelonephritis, and other diseases that impair the renal function.^10^^;^^11^^;^^12^ Currently, a variety of minimally invasive surgeries have been available to treat ureteral calculi,^13^ but some patients develop postprocedural complications that seriously disturb their daily life, aggravate their disease burden, and even trigger stress responses, thus preventing the patient from following medical orders for rehabilitation and prolonging the hospitalization time.^14^ The routine nursing methods can hardly improve patient psychological state, leading to poor recovery, longer than expected recovery time, and achieving suboptimal therapeutic effects.^15^

This study showed that the “five‑in‑one” mode for assisting in medical treatment can hugely improve patient physical and mental status, alleviate pain, reduce the incidence of postoperative complications, and enhance the compliance and satisfaction with nursing. This method creates an optimal recovery environment for patients, while ensuring the advancement of therapeutic methods and stabilization of mental status. It enables the patients to participate in the rehabilitation treatment more actively, and to meet the patient demands as much as possible by guaranteeing the treatment efficiency^16^ It is speculated that pain relief along with improvement in mental and physical status and satisfaction with nursing are the elements of the multifaceted supervision that enable the patients to fully comply with the rehabilitation training and to improve their physical condition. In the meantime, the nurses and the patient family members communicate with the patients, and their encouragement safeguards the mental status of the patients. The meditation and attention distraction methods taught by the therapists reduce the feeling of pain, the reasonable rehabilitation scheme formulated by the doctors lowers the probability of complications, and the long‑term communication makes the patients more intimate with the medical staff, finally resulting in an increase in the patient satisfaction with nursing.^17^

We identified a number of important reasons for the improved patient compliance. First, the medical staff have spent a long time with the patients and were familiar with them, which enhanced the patient compliance. Second, the medical staff had more interactions with the patients, and the patients understood the significance of their own behaviors, which improved their compliance. Third, the encouragement from both medical staff and family members improved the mental status of the patients, thereby strengthening their compliance.^18^

The decrease in complication rate was probably due to the reasonable rehabilitation scheme developed by the doctors (perfectly executed by the patients under the coordinated supervision of all aspects, thus avoiding complications due to irregular behaviors of the patients), active nursing by the nurses (they can communicate to understand patient condition over time and take measures to reduce the complications caused by the untimely discovery of problems), the rehabilitation training developed by the therapists (to restore mobility as early as possible and prevent complications due to prolonged bed rest), and the interaction between the patient family members and the patients (to reduce the tension and prevent complications induced by negative emotions).^19^^;^^20^^;^^21^

Nevertheless, this study has some limitations. The sample size was small, and the study period was short. Additionally, it was a single‑center study. Further multicenter studies with larger sample size are needed to prove our findings.

## CONCLUSIONS

In conclusion, the “five‑in‑one” mode can stabilize the mental status, ameliorate the QoL and satisfaction with nursing, reduce negative emotions, decrease the complication rate, and improve the compliance of patients.
